# Dinner to Dr. Pryor

**Published:** 1904-04

**Authors:** 


### Dinner to Dr. Pryor.

DR. JOHN H. PRYOR, of Buffalo, who has been appointed superintendent of the New York State Hospital for the treatment of patients suffering from incipient tuberculosis, located at Raybrook, Essex county, in the Adirondack region, was tendered a dinner by a group of his friends who are members of the Saturn Club. The banquet was served at the club on the evening of March 17, 1904, and was presided over by Dr. Stephen Y. Howell. Besides Dr. Pryor, who was the guest of honor, the following-named gentlemen were present: J. N. Adam, Francis Almy, Frederick Almy, Louis L. Babcock, John H. Bell, Walter L. Brown, Dr. L. Burrows, Clarence W. Cady, Newcomb Carlton, Martin Clark, Edward W. Dann, Jesse C. Dann, William A. Douglas, Joseph G. Dudley, W. Allen Gardner, Edward B. Guthrie, H. S. Guthrie, Dr. H. E. Hayd, George Harrower, Charles B. Hill, Dr. Stephen Y. Howell, Dr. Arthur W. Hurd, William B. Hull, Dr. Charles S. Jewett, Dr. F. Park Lewis, Shumway Lee, Frank M. Loomis, Dr. H. G. Matzinger, H. G. Meadows, Knowlton Mixer, Austin K. Muzzey, John A. Mann, John B. Olmstead, Dr. Roswell Park, Dr. John Parmenter, Dr. James W. Putnam, R. W. Pomeroy, Dr. W. S. Renner, Dr. DeLancey Rochester, Harry Rumrill, John Sedgwick, Henry W. Sprague, Dr. P. W. Van Peyma, Frederick A. Vogt, H. C. Wadsworth, General S. M. Welch, Dr. Ernest Wende, Ansley Wilcox, Arthur H. Williams, Harry D. Williams, Dr. H. U. Williams, John L. Williams.

A loving cup was presented to Dr. Pryor, John B. Olmstead, Esq., acting as spokesman in behalf of the donors. In the issue of the JOURNAL for March, 1904, the fact was mentioned that Dr. Pryor had passed the state civil service examination, and had become eligible for this appointment. It appears that Dr. Pryor was prevailed upon to accept the appointment by his former associates upon the board of trustees. His superior fitness for the place, manifested during several years work upon the board, was recognised. Dr. W. G. Macdonald, of Albany, one of the trustees, is quoted as voicing the opinion of the board in the following language:

Dr. Pryor is regarded peculiarly as an expert on diseases of the chest. He has so been regarded throughout the country for a number of years. He is the pioneer advocate of state care of incipient tuberculosis, and his experience in developing that idea equips him with special qualifications for this position.

He has been connected for a number of years with the large hospitals of Buffalo, and was instrumental in having the Erie County Hospital provide a department for the care of tubercular patients. The board of trustees regards itself as being peculiarly fortunate in securing the services of Dr. Pryor. The appointment may be characterised as advantageous to the state and it is not without personal sacrifices upon the part of Dr. Pryor. For a number of years he has been associated with propositions which the state has now practically determined to solve, and it could find no better agent nor no more conscientious observer than Dr. Pryor.

While Dr. Pryor's countless friends in Buffalo will regret his departure from the city, all recognise the wisdom of his selection to perform the duties of this important state institution and unite in wishing him the best success.

American Medicine is making a splendid fight against the medical advertiser in the daily newspapers, and in a recent issue editorially calls attention to the use of the mails by the newspapers containing fraudulent and filthy advertisements. An especially flagrant case of foulness is thus referred to:

The columns of the most popular papers, both of the city and county, are reeking horrors, although they are said to be "family" journals, appearing at the breakfast table for the instruction, not only of the men, but of mother, daughter, and child. And they are allowed the benefits of the United States Mail Service, although fraud and deception are rampant in them and crime is encouraged. Why will any decent person, for example, buy or allow the *New York Sun* of Sunday, February 21, to come into his house? Prominent in its columns is a most nauseating picture advertisement headed: "Unhappy Homes Caused by Weakness in Men." Even in a medical journal we hesitate to repeat the disgusting parts of the advertisement about the "shrunken organs," etc., and copy only the last paragraph, which runs as follows:

The lucky discoverer simply desires to get in touch with all men who can make use of such a treatment. They should address him in confidence, Dr. H. C. Raynor, 339 Luck Building, Detroit, Mich., and immediately on receipt of your name and address, it is his agreement with this paper to send you a free receipt or formula of this modern treatment by which you can cure yourself at home.

Note the words, "his agreement with this paper."

---

Prior to the appointment of the Panama Canal Commission the committee on Medical Legislation of the American Medical Association, Dr. Charles A. L. Reed, of Cincinnati, chairman, urged that one of the members of that commission be a medical man entrusted with the work of sanitation. Attention was called to the frightful mortality on the canal work under French regime, and it was urged that the highest administrative authority be invested in the appointment suggested. The name of Dr. Gorgas, whose brilliant work in Cuba has made for him a lasting reputation, was mentioned later in connection with the appointment. The commission has been announced and there does not appear among the names that of any man whose knowledge of sanitation is more than purely superficial or any deeper than is absolutely necessary to an engineering education. The only remedy is for the army medical department to be placed in control of the medical situation on the Isthmus. Then there will be results just as there were results in Cuba and the Philippines when the army took charge.

A TRAINED nurse, Miss Alt, slipped on the icy sidewalk at Elmwood avenue and North street, Buffalo, Sunday night, February 28, and fractured one of her legs. She was assisted to the steps of a house in front of which a carriage was waiting. A passer-by rang the bell and asked if the carriage might take the suffering woman to her home, a short distance away. Permission was refused, because the residents of the house wanted to go to church in the carriage, and Miss Alt was allowed to sit on the steps in water-soaked clothing without the slightest offer of assistance from the people in the house, which is occupied by the first reader of a christian science church, who swept by Miss Alt without so much as looking at her. That is Eddyism or christian science. Across the street lives Mr. George B. Hayes. His son came from the house to mail a letter; he saw the injured woman and went to her with offers of shelter and assistance. That's humanity. And there is a wide difference between christian science and humanity. One is a selfish, narrow-minded fad, the other is real Christianity.

---

AN ASSOCIATED press dispatch, dated March 21, 1904, indicates that another step has been taken toward the union of the State Medical Society and Association. It reads as follows: "Tonight the New York State Medical Association went out of existence and, by a resolution unanimously adopted, took the last action necessary to complete the consolidation of the two state medical organizations which have occupied the field in opposition for more than twenty years. The other society whose name will be retained by the new organisation, the Medical Society of the State of New York, has already taken necessary action."

---

OUR readers are well aware that the JOURNAL rarely speaks of its own affairs in its columns. Recently, however, two compliments from excellent sources have come to our hands as such agreeable surprises that we fain would mention them. One, which is from the *Buffalo Evening News*, is referred to elsewhere. The other reads as follows:

KRESS & OWEN COMPANY,
Manufacturing Chemists,
210 Fulton street, New York,
March 11, 1904.

BUFFALO MEDICAL JOURNAL,
William Warren Potter, M. D., Editor.

*Dear Doctor*—We desire at this time to express our sentiments of appreciation due your journal. As an advertising medium it has well served its part in the establishing of our product, glycothymoline. How well we have succeeded may be mirrored in the statement of a prominent New York clinician, who recently remarked, "glycothymoline in the treatment of catarrhal conditions of mucous membranes has become as standard as any drug in the pharmacopeia."

Thanking you for any interest in our behalf and wishing a most prosperous season for your JOURNAL, we remain,

Yours very truly,

KRESS & OWEN COMPANY,
SAMUEL OWEN,
President.

Thank you, gentlemen, and please accept our cordial appreciation of your kind words.

---

